# Normalizing Google Scholar data for use in research evaluation

**DOI:** 10.1007/s11192-017-2415-x

**Published:** 2017-05-22

**Authors:** John Mingers, Martin Meyer

**Affiliations:** Kent Business School, University of Kent, Canterbury, UK

**Keywords:** Google Scholar, Normalization, Research evaluation

## Abstract

Using bibliometric data for the evaluation of the research of institutions and individuals is becoming increasingly common. Bibliometric evaluations across disciplines require that the data be normalized to the field because the fields are very different in their citation processes. Generally, the major bibliographic databases such as Web of Science (WoS) and Scopus are used for this but they have the disadvantage of limited coverage in the social science and humanities. Coverage in Google Scholar (GS) is much better but GS has less reliable data and fewer bibliometric tools. This paper tests a method for GS normalization developed by Bornmann et al. (J Assoc Inf Sci Technol 67:2778–2789, 2016) on an alternative set of data involving journal papers, book chapters and conference papers. The results show that GS normalization is possible although at the moment it requires extensive manual involvement in generating and validating the data. A comparison of the normalized results for journal papers with WoS data shows a high degree of convergent validity.

## Introduction

The evaluation of research performance is becoming ever more common, whether at the level of the individual academic, the department or institute, or the university or multiversity (Gingras 2016). Although much of this is judgement-based in the form of peer review, the use of bibliometric data is also becoming more common although there is debate as to whether citations are indicators of quality or impact (Leydesdorff et al. 2016). There are two main sources of citations—specialized databases such as *Web of Science* (WoS) or *Scopus*, and *Google Scholar* (GS) which searches the web to find citations from different sources. There have been many comparisons of the relative advantages and disadvantages of these sources (Adriaanse and Rensleigh 2013; Crespo et al. 2014; Harzing and Alakangas 2016; Meho and Yang 2007; Mingers and Lipitakis 2010; Prins et al. 2016).

The main conclusions of these comparisons are that WoS and Scopus generally provide robust and accurate data for the journals that they cover, and that they also provide significant extra functionality including journal lists relating to particular fields. But, there are significant limitations in terms of their coverage of the non-science disciplines. Studies have shown (Amara and Landry 2012; Mingers and Lipitakis 2010) that in social science often less than 50% of the publications of a person or institution actually appear in the database and the numbers of citations of those that are included are correspondingly lower. In arts and humanities, where much of the research output is in the form of books rather than papers, the situation is very much worse. This has led several commentators to conclude that bibliometrics cannot be used in these fields at the moment (Van Leeuwen 2013; Wilsdon et al. 2015).

In contrast, GS has significant problems of data reliability and validity but has a much better coverage of social science and humanities research—in fact it has the same level of coverage as for the sciences. Martín-Martín et al. (2014) claim that GS now sweeps almost the entire academic web—publishers, digital hosts, scholarly societies, disciplinary databases, institutional repositories and personal webpages. This makes it potentially a valuable resource for evaluation in these areas (Bornmann et al. 2016; Harzing 2013, 2014; Prins et al. 2016).

However, one problem with GS is that of normalization. Citation rates differ markedly (by orders of magnitude) between different fields with the sciences, and especially medicine and biology, having much greater citation rates than social science. This means that any form of comparison between different fields should be done on the basis of data that has been normalized to the field in some way (Bornmann and Marx 2015; Leydesdorff et al. 2011; Opthof and Leydesdorff 2010; Waltman and van Eck 2013). There are several approaches to normalization, but the most common involves comparing the citations received by papers under review to citations received by papers published in the same journal or the same field as a whole (Leydesdorff and Opthof 2011; Moed 2010a; Opthof and Leydesdorff 2010; Waltman et al. 2010, 2011).

Normalization has conventionally only been applied to WoS and Scopus both because of the greater reliability of the data, and because of the availability of field lists of journals in WoS and this has limited the extent to which GS has been used in research evaluation. However, recently two studies have tried to apply normalization to GS data. Prins et al. (2016) compared WoS and Google Scholar in a study of the fields of education and anthropology in Holland. In the paper they say that they tried to normalize the GS data using the interface *Publish or Perish (*PoP) (Harzing 2007) and that the results were technically feasible but rather unsatisfactory. No further information was given. Bornmann et al. (2016) conducted a more explicit test using data on 205 outputs from a research institute. Of these, 56 were papers also included in WoS, 29 papers not covered by WoS, 71 book chapters, 39 conference papers and 10 books.

In this paper we aim to generally follow the approach of Bornmann et al. (2016) and test the method on a sample of outputs from the business and management field. The first section outlines the data and methods used and the second provides the results for a selection of journal papers, book chapters and conference papers.

## Methods

### Data

As data for this research we have chosen all the publications of one of the authors (Mingers). Although this may seem unusual, this approach has been employed before by Harzing (2016) and does have a number of advantages:

1. There are a significant number of publications (see Table 1) nearly all of which are well-cited, with over 10,000 GS citations in all. Of the journal papers, all were found in GS but only 73% are included in WoS. There are also book chapters and conference papers.

**Table 1:** Outputs in the dataset

Output type	Number
Refereed journal papers in Google Scholar	85
Refereed journal papers in Google Scholar and WoS	62
Book chapters	17
Conference proceedings	15
Books	7
2. The publications cover a long time period (1980–2013) so that any time-based effects may be noticed.
3. They cover a wide range of journals in a variety of fields—operational research, information systems, bibliometrics, systems thinking, philosophy and sociology. Many are in leading journals such as *MIS Quarterly* and *European Journal of Operational Research,* while some are in quite obscure, niche journals.
4. Because they are the author’s papers, the exact publication details are known. This is particularly important for conference papers, which are often difficult to pin down with somewhat scanty details, and for book chapters in terms of finding all the other chapters in the book.

### Normalization

There are two main forms of normalization (Leydesdorff et al. 2011; Waltman et al. 2013)—cited-side normalization (Mingers and Lipitakis 2013; Opthof and Leydesdorff 2010; Waltman et al. 2010, 2011) or citing-side (source normalization) (Moed 2010b; Waltman et al. 2013; Zitt 2010, 2011). The former compares a paper’s citations to the number of citations received by other, similar papers. Examples are the journal normalized citation score (JNCS) and the mean (field) normalized citation score (MNCS). The latter compares them to the source of citations—reference lists in the citing papers—an example being source normalized impact per paper (SNIP). Bornmann and Haunschild (2016) have suggested a combination in which the citations are normalized with respect to the cited-side number of references. There are other forms of normalization, for example normalizing for the number of authors can be done in PoP (Harzing et al. 2014), but these will not be considered here.

The problem with source normalization is that it is not possible for the ordinary researcher as it requires complete access to a database such as Scopus or WoS and software to carry out the searches (Leydesdorff and Opthof 2010a, b). It would not be possible with GS because GS limits access especially from robotic searches. We will therefore use cited-side normalization and, in particular, journal as opposed to field normalization, as did Bornmann et al. (2016). This is because there are no field lists of journals available in GS.

The JNCS is defined as follows:

The number of citations to each of the unit’s publications is normalized by dividing it with the world average of citations to publications of the same document type, published the same year in the same journal. The indicator is the mean value of all the normalized citation counts for the unit’s publications” (Rehn et al. 2007, p. 22).

The traditional way to calculate MNCS or JNCS according to the Leiden methodology (Waltman et al. 2010, 2011) was to total the actual citations and the expected (average) citations of a set of papers and then divide the two. However, Leydesdorff (2011) and Opthof and Leydesdorff (2010) pointed out that mathematically this was the incorrect order and that it would bias the results towards the papers with larger numbers of citations. Instead, they argued that the JNCS or MNCS should be calculated individually for each paper and then these should be averaged. This was accepted by Leiden (Waltman et al. 2010, 2011).

In order to operationalize this, it is necessary to find the citations of the paper in question and then find all the citations to papers of the same type that were published in the same journal and year which is the complex part, especially with GS. We then calculate the average citations per paper (CPP) for the journal and divide that into the citations for the target paper to give the normalized citations for the paper. A value of 1 means that the paper is cited at an average rate for that journal and year; a value of more (less) than 1 means that it is more (less) highly cited than average. The mean of all the normalized citations is then the JCNS for the person or institution.

We should note that, while WoS allows the type of paper (article, review, note, editorial) to be a criteria, GS does not. Thus when we calculated the JNCS’s from WoS we specified type as article or review, but with GS we were not able to do this. We do not believe this has affected the results much, if there is an effect it would be to increase the JNCS for WoS since other types of papers, such as editorial or book reviews, are generally cited less.

For book chapters and conference papers, the procedure is the same except that the output is normalized to the relevant book or conference that the output is part of. Searches were carried out using both GS and PoP and specific search procedures are discussed below.

## Results

### Journal papers

The first stage is finding the number of citations for a particular paper. It is relatively easy as there are a range of search terms available. Generally, the name, year and title find the correct paper. Sometimes there are different versions as they have been mis-cited in references. It would require a judgement as to whether or not to accumulate the citations of the variants into the total. Another peculiarity of GS that occurs sometimes is that the paper appears when searched for individually but does not appear in the list of papers published by that journal in the year. For the searches we used both GS itself and also PoP and we often had to try different search strings to generate reasonable results—examples will be discussed later.

Looking up the number of citations that a paper has received is generally straightforward with author and title usually sufficing. It is harder, however, to find all the papers and citations for the whole journal in the appropriate year. Consider a randomly-chosen example—the journal *Management Learning* in 2010. The different results for various search term are shown in Table 2. The actual count of papers from the journal website is 30 plus 28 book reviews (which would not be included here because they are not the correct type of paper). Using just the name of the journal generates 682 papers; putting it in quotes reduces that to 155. However, many of these are from other journals such as *Academy of Management Learning and Education* which share part of the name. Unfortunately, in GS, using quote marks round the name does not restrict results to exclusively that name. Putting “academy” as an exclusion reduces the count to 30.

**Table 2:** Numbers of papers and citations for different search combinations for the journal “Management Learning”, 2010 from PoP. Exactly the same results were found from direct GS searches

Source	Search terms	Papers	Citations	CPP
Actual number from journal website		30 + 28 book reviews		
PoP	Management Learning	682	5896	8.6
	“Management Learning”	155	4202	27.1
	“Management Learning” with ISSN	62	1386	22.4
	“Management Learning” excluding “Academy”	30	169	5.6
	ISSN	71	1494	21.0
	ISSN excluding “Academy”	29	243	8.6

On some occasions, these search modalities still left spurious entries that had to be removed by hand. One extreme example was a paper in the Journal of the Operational Research Society 2002 that appeared to have over 10,000 citations by itself. It turned out to be a book review which had inherited the citations of the book. Another journal—*Journal of Information Technology*—had a name that was extremely common being part of over twenty other journal names. In this case, including the publisher as a search field helped significantly. A further particular problem was journals that have “&” in their title as they are often spelt with “and” in citations. This can be dealt with searching for both titles using “OR”.

Whilst not all journals have this many problems—many get the approximately correct number straight away—it does nevertheless mean that there needs to be manual checking each time, it cannot be an automated process.

The overall results for journals are shown in Table 3 which shows GS results for both all papers and only those also included in WoS. We can see that the mean citation per paper (CPP) is much higher for GS (about 3 times) as is commonly found (Mingers and Lipitakis 2013). But, despite the difference in absolute numbers of citations, the JNCS’s are actually very similar—2.65 in GS compared with 2.53 in WoS. Given the wide range of journals and the long timespan this indicates a high degree of convergent validity.

**Table 3:** Summary results for journals

	GS journal paper citations (all)	GS journal paper citations (only papers in WoS)	WoS journal paper citations	GS JNCS (all)	GS JNCS (only papers in WoS)	WoS JNCS
Arithmetic mean	104.92	100.6	33.69	2.78	2.65	2.53
Median	44	48	18	2.02	1.91	1.65
*n*	85	62	62	85	62	62

**Fig. 1:**
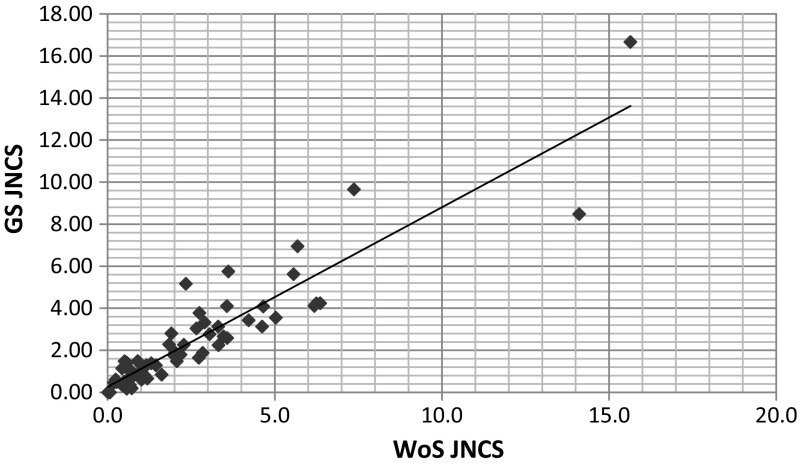
Scattergram of WoS JNCS against GS JNCS with linear regression line

More revealing is Fig. 1 which shows a scattergram of the WoS and GS JNCS’s together with a linear regression. As can be seen, the two datasets correlate very well (*r* = 0.94) and the slope of the regression is close to 1 as would be hoped for (*b* = 1.04, *t* = 19.72). Nor are there significant outliers which would show that certain papers had very different results under the two systems.

### Book chapters

Within the dataset there were 17 book chapters in 13 books. All but one of the books were found in GS, the one being a translation in Slovenian. Getting full information required considerable manual intervention. The search strategy involved looking up the book title using the “published in” and “year” fields in either GS or PoP. This generally resulted in most but not all of the chapters, together with incorrect or duplicate results. It was therefore necessary also to look up the book on the internet in order to find out the actual chapters that it contained. Then these could be searched for individually to ensure that all occurrences were found. It was often difficult to find individual chapters and a variety of searches were employed. If it was impossible to find a chapter in GS it was ignored although arguably it could have been included with zero citations.

The results are shown in Table 4 and a summary in Table 5. The overall BCNCS was 2.17 which is not dissimilar from the JNCS.

**Table 4:** Actual results for book chapters

Chapter code	Citations of the chapter	Chapters found in book	Total citations for the book chapters	Citations per chapter in book	BCNCS
104	3	25	120	4.80	0.63
105	99	11	678	61.64	1.61
106	35	15	307	20.47	1.71
107	46	12	439	36.58	1.26
108	97	14	536	38.29	2.53
109	14	14	536	38.29	0.37
110	11	14	536	38.29	0.29
111	125	14	536	38.29	3.26
112	10	12	78	6.50	1.54
113	14	22	75	3.41	4.11
114	36	16	87	5.44	6.62
115	134	12	544	45.33	2.96
116	166	15	860	57.33	2.90
117	153	15	860	57.33	2.67
118	40	9	207	23.00	1.74
120	96	10	1559	155.90	0.62

**Table 5:** Summary results for book chapters

	GS chapter citations	GS number of chapters	GS total chapter citations	Citations per chapter	BCNCS
Arithmetic mean	67.44	14.38	497.4	39.43	2.17
Median	43	14	536.3	38.29	1.72
*n*	16	16	16	16	16

### Conference papers

Conference papers proved to be the hardest to deal with. The first problem is finding all the papers from the conference. This is because there are many possible reference names for the conference and search terms that can be used. For example, there is a yearly conference organized by the International Association of Engineers (IAENG) which is called the *International Multiconference of Engineers and Computer Scientists (IMECS)*. Searching for the 2011 conference using the full title received zero hits. Searching for IMECS 2011 in the “Publication” field received 13 hits. Searching for “IMECS 2011 Proceedings” in the “Exact phrase” field received no hits, but searching for “IMECS 2011” in the “Exact phrase” field received 331 hits, many although not all of which were relevant. But this pattern was not consistent across conferences. For example, the *International Conference on Information Systems* received a reasonable number of hits with both “ICIS 2008” and “ICIS 2008 Proceedings” in both the “Publication” field (210 and 202) and the “Exact phrase” field (290 and 230) although there were many false entries in the latter. In the end, details of two conferences could not be found at all. We did attempt to look up the conferences in WoS but this generally did not work. There appears to be no list available of conferences that WoS covers with the titles it uses.

There was also a problem on the other side in finding the specific paper that was being evaluated. Sometimes it would not appear in the list of conference papers and would not appear even if it was searched for directly by title/author/year. This may happen when there is a later version of the paper that has been published in a journal and all the different variants get swept up into that. In one instance the conference paper could not be found by itself but it did appear in the listing of “all versions” of the corresponding journal paper.

The overall results for the conferences are shown in Table 6, and the summary in Table 7. The CPNCS for the conferences that were found was 1.25, significantly lower than that of book chapters and journals, but this was quite a small sample and there was considerable variation including those papers that were not found. If the conferences where the paper was not found are excluded the CPNCS goes up to 2.17 which is closer to the other types of publication.

**Table 6:** Actual results for conference papers

Paper code	Citations of the conference paper	Papers found in conference	Total citations for the conference papers	Citations per conference paper	CPNCS
123	0	0	0	0.00	0.00
124	40	0	19	2.11	0.00
125	1163	0	256	4.54	0.00
126	1995	0	195	10.23	0.00
127	990	13	89	11.12	1.17
128	1600	2	79	20.25	0.10
129	95	2	52	1.83	1.09
130	3001	13	410	7.32	1.78
131	130	3	157	0.83	3.62
132	19	3	23	0.83	3.63
133	19	0	23	0.83	0.00
134	0	0	0	0.00	0.00
135	240	5	129	1.86	2.69
136	1428	17	28	51.00	0.33
137	310	14	106	2.92	4.79

**Table 7:** Summary results for book chapters

	GS conference paper citations	GS number of papers	GS total conference citations	Citations per conference paper	CPNCS
Arithmetic mean	4.8	104.4	735.33	7.71	1.28
Median	2	240	79	2.10	0.33
*n*	13	13	13	13	13

### Books

There are seven books in the dataset, four research monographs and three edited collections. The earliest is from 1994 and the most recent from 2014. All of these were found in GS with citations numbers ranging from 23 (for the most recent) to 1534. However, at the moment there is no method of normalizing a book’s citations and it is difficult to see how a field or domain of appropriate books for normalization could be specified. A possible approach through citing-side normalization could be envisaged, as the domain would be all the books (rather than papers, presumably) that cited the book in question. The problem there would be counting all the references within the citing books.

Some books are now included in WoS in the Book Citation Index (BCI) but Bornmann et al. (2016) and Torres-Salinas et al. (2014) concluded that it is not yet sufficiently well developed to be useful for citation analysis or normalization.

## Discussion

Google Scholar provides a very valuable resource for bibliometric analysis and for using citations in the evaluation of research. It has clear advantages over WoS and Scopus in terms of its coverage of the social sciences and humanities. It also covers forms of research output other than journal papers such as books, book chapters and conference papers. However, all citation-based analysis needs to be normalized to its research field and this has generally been carried out either in WoS (cited-side normalization) of Scopus (citing-side normalization). In this paper we have investigated the possibility of normalizing GS data.

The main conclusions are that it is indeed possible to normalize papers, book chapters and conference papers although not, at this point, books. The citations for papers could be triangulated with WoS data and the results showed a high degree of convergence despite the differences in coverage between the two, and the much greater level of citations in GS. Normalized results could also be obtained for chapters and conference proceedings although they could not be triangulated.

The main limitation of this approach is the large amount of manual intervention that is necessary. In all three areas, but especially in conference proceedings, several different approaches had to be used to find the relevant reference papers, and much erroneous material was produced which had to be removed by hand. Even after this, the data was far from complete and accurate. Nevertheless, the overall results show that much of this noise is irrelevant in terms of the highly aggregated normalized results that were produced. Other problems with GS data include the possibility of manipulating GS indicators (Delgado López-Cózar et al. 2014) and the lack of stability of the data over time (Martín-Martín et al. 2014).

Many of the problems with GS arise, not because of the underlying searching and data collection, but because of the interface which allows the user very little control over the presentation of the results, and the difficulty of accessing the data in an automated fashion. Presumably this is because GS is designed simply to present data to users who want to find relevant papers in an easy way; it has not been designed as a serious bibliometric tool. Perhaps there is an opportunity for *Google* to provide such an interface to the data which institutions would be prepared to pay for.

In terms of limitations of this paper, the dataset is fairly small, especially in terms of book chapters and conference papers and a much larger set would be valuable, although the analysis of it would be extremely time-consuming. The other limitation is that the form of normalization is to only the journal, conference or book. It would be more satisfactory to normalize to a wider domain or field but reference sets to do this are not readily available.

## References

[CR1] Adriaanse L, Rensleigh C (2013). Web of science, scopus and Google Scholar. The Electronic Library.

[CR2] Amara N, Landry R (2012). Counting citations in the field of business and management: Why use Google Scholar rather than the web of science. Scientometrics.

[CR3] Bornmann L, Haunschild R (2016). Citation score normalized by cited references (CSNCR): The introduction of a new citation impact indicator. Journal of Informetrics.

[CR4] Bornmann L, Marx W (2015). Methods for the generation of normalized citation impact scores in bibliometrics: Which method best reflects the judgements of experts?. Journal of Informetrics.

[CR5] Bornmann L, Thor A, Marx W, Schier H (2016). The application of bibliometrics to research evaluation in the humanities and social sciences: An exploratory study using normalized Google Scholar data for the publications of a research institute. Journal of the Association for Information Science and Technology.

[CR6] Crespo JA, Herranz N, Li Y, Ruiz-Castillo J (2014). The effect on citation inequality of differences in citation practices at the web of science subject category level. Journal of the Association for Information Science and Technology.

[CR7] Delgado López-Cózar E, Robinson-García N, Torres-Salinas D (2014). The Google Scholar experiment: How to index false papers and manipulate bibliometric indicators. Journal of the Association for Information Science and Technology.

[CR8] Gingras Y (2016). Bibliometrics and Research Evaluation: Uses and Abuses.

[CR14] Harzing, A.-W. (2007). Publish or Perish. http://www.harzing.com/pop.htm.

[CR9] Harzing A-W (2013). A preliminary test of Google Scholar as a source for citation data: a longitudinal study of Nobel prize winners. Scientometrics.

[CR10] Harzing A-W (2014). A longitudinal study of Google Scholar coverage between 2012 and 2013. Scientometrics.

[CR11] Harzing A-W (2016). Microsoft academic (search): a phoenix arisen from the ashes?. Scientometrics.

[CR12] Harzing A-W, Alakangas S (2016). Google Scholar, scopus and the web of science: a longitudinal and cross-disciplinary comparison. Scientometrics.

[CR13] Harzing A-W, Alakangas S, Adams D (2014). hIa: An individual annual h-index to accommodate disciplinary and career length differences. Scientometrics.

[CR15] Leydesdorff, L., Bornmann, L., Opthof, T., & Mutz, R. (2011) Normalizing the measurement of citation performance: Principles for comparing sets of documents,” arXiv.

[CR16] Leydesdorff, L., Bornmann, L., Comins, J., & Milojević, S. “Citations: Indicators of quality? The impact fallacy,” *arXiv preprint* arXiv:1603.08452) 2016.

[CR17] Leydesdorff L, Opthof T (2010). Scopus’s source normalized impact per paper (SNIP) versus a journal impact factor based on fractional counting of citations. Journal of the American Society for Information Science and Technology.

[CR18] Leydesdorff L, Opthof T (2010). Scopus SNIP indicator: Reply to Moed. Journal of the American Society for Information Science and Technology.

[CR19] Leydesdorff L, Opthof T (2011). Remaining problems with the “New Crown Indicator” (MNCS) of the CWTS. Journal of Informetrics.

[CR20] Martín-Martín, A., Orduña-Malea, E., Ayllón, J. M., & López-Cózar, E. D. “Does Google Scholar contain all highly cited documents (1950–2013)?,” *arXiv preprint* arXiv:1410.8464) 2014.

[CR21] Meho L, Yang K (2007). Impact of data sources on citation counts and rankings of LIS faculty: Web of Science, Scopus and Google Scholar. Journal American Society for Information Science and Technology.

[CR22] Mingers J, Lipitakis E (2010). Counting the citations: A comparison of Web of Science and Google Scholar in the field of management. Scientometrics.

[CR23] Mingers J, Lipitakis E (2013). Evaluating a department’s research: Testing the leiden methodology in business and management. Information Processing and Management.

[CR24] Moed H (2010). CWTS crown indicator measures citation impact of a research group’s publication oeuvre. Journal of Informetrics.

[CR25] Moed, H. (2010b) The Source-Normalized Impact per Paper (SNIP) is a valid and sophisticated indicator of journal citation impact, In: *arXiv preprint*, arxiv.org.

[CR26] Opthof T, Leydesdorff L (2010). Caveats for the journal and field normalizations in the CWTS (“Leiden”) evaluations of research performance. Journal of Informetrics.

[CR27] Prins, A. A. M., Costas, R., van Leeuwen, T. N., & Wouters, P. F. (2016) Using Google Scholar in research evaluation of humanities and social science programs: A comparison with Web of Science data, *Research Evaluation*, February 2.

[CR28] Rehn C, Kronman U, Wadskog D (2007). Bibliometric indicators—Definitions and usage at Karolinska Institutet.

[CR29] Torres-Salinas D, Robinson-Garcia N, Miguel Campanario J, Delgado Lopez-Cozar E (2014). Coverage, field specialisation and the impact of scientific publishers indexed in the book citation index. Online Information Review.

[CR30] Van Leeuwen, T. (2013). Bibliometric research evaluations, web of science and the social sciences and humanities: A problematic relationship? *Bibliometrie*-*Praxis und Forschung,* (2), 8-2–8-18.

[CR31] Waltman L, van Eck N (2013). A systematic empirical comparison of different approaches for normalizing citation impact indicators. Journal of Informetrics.

[CR32] Waltman L, van Eck N, van Leeuwen T, Visser M (2013). Some modifications to the SNIP journal impact indicator. Journal of Informetrics.

[CR33] Waltman L, van Eck N, van Leeuwen T, Visser M, van Raan A (2010). Towards a new crown indicator: Some theoretical considerations. Journal of Informetrics.

[CR34] Waltman L, van Eck N, van Leeuwen T, Visser M, van Raan A (2011). Towards a new crown indicator: An empirical analysis. Scientometrics.

[CR35] Wilsdon, J., Allen, L., Belfiore, E., Campbell, P., Curry, S., Hill, S., et al. (2015). The metric tide: Report of the independent review of the role of metrics in research assessment and management, HEFCE, London.

[CR36] Zitt M (2010). Citing-side normalization of journal impact: A robust variant of the Audience Factor. Journal of Informetrics.

[CR37] Zitt M (2011). Behind citing-side normalization of citations: Some properties of the journal impact factor. Scientometrics.

